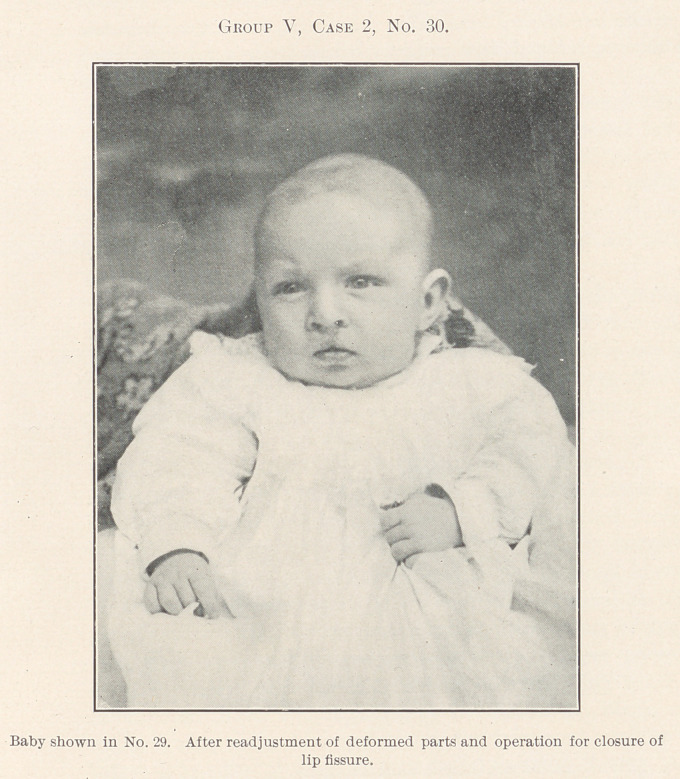# The Vital Significance of Oral Disease

**Published:** 1905-05

**Authors:** G. V. I. Brown

**Affiliations:** Milwaukee, Wis.


### International Dental Journal.

Vol. XXVI. May, 1905. No. 5.

### Original Communications.[1]

### THE VITAL SIGNIFICANCE OF ORAL DISEASE.[2]

BY DR. G. V. I. BROWN, MILWAUKEE, WIS.

It is my purpose this evening to use the term vital both in its meaning as being essential and in relation to life, but more especially the latter.

That the work of the oral therapeutist is essential to the health and well-being of the individual goes without saying; that it has a more or less important influence upon pathologic conditions of other parts of the organism, and *vice versa*, is also generally admitted, yet the true relation with regard to vitality is not fully appreciated.

In recent years the great work Talbot has done in the study of auto-intoxication as applied to oral disease and constitutional causes of dental caries has begun to be better understood in its true significance.

Kirk's recognition of certain forms of crystallization in the salivary secretion, and the study of these in association with pathologic expression of disease in other parts, has opened a vast field potent with great possibilities, which we trust may be developed into true science and adopted as a part of diagnosis.

[1] The editor and publishers are not responsible for the views of authors of papers published in this department, nor for any claim to novelty, or otherwise, that may be made by them. No papers will be received for this department that have appeared in any other journal published in the country.

[2] Read before The New York Institute of Stomatology, January 2, 1905.

It is believed the slides this evening will demonstrate both the value and the absolute necessity of giving full consideration to the vital interrelation between general conditions of health and anatomical construction and the tissues of the mouth in dealing with diseases and deformities thereof, and will be in the nature of a practical demonstration, since all of the illustrations are from cases in my own practice.

The several groups of pictures are introduced to show—

1. Conditions of the blood and visceral organs coincident with disease of the maxillary bones and mucous membrane of the mouth.

2. The value of a recognition of first principles, or the true etiology in the correction of these ills.

3. Extreme nervous affections due to, or at least associated with, dental or oral disturbance.

4. Some of the predisposing and exciting causes of malnutrition, local as well as general.

5. The effect of prenatal and postnatal arrest of development upon mouth, nose, and face, and these in turn on the human organism as a whole.

6. The bearing of all this upon longevity.

Necessarily the principles upon which the theoretical structure whereon rests the practical deductions that led to the adoption of the methods of treatment, and results shown, cannot of themselves be new; questions of assimilation, nutritional function, physiologic efficiency of the dental organs as part of the whole digestive system, nerve distribution and its possibilities of reflex action, are matters of common knowledge in an audience such as this, and, moreover, have been the theme of so many, and such profuse writing, as to be of little interest so far as general features are concerned.

We are accustomed to be told that affections of the teeth and jaws are frequent causes of remote, or even serious pathologic disturbance, and that it is sometimes difficult or impossible to effect a cure in the face of ill health, be it general or directed to some special organ. But to-night we are brought face to face with death; the screen will show us lesions that no therapeutic or surgical power at present under our command can control. We will pass for the moment beyond that which our usual consideration contemplates, and face actual conditions that are, as a rule, only hinted at or casually referred to by essayists, and look upon real conditions which I believe have not before been shown with absolute certainty of detail in the definite manner we shall undertake to view them for our present purpose, and yet which, notwithstanding the infrequency of actual demonstration, are everywhere about us in one form or another, to be seen if only their diagnosis were better understood.

GROUP I.—Case 1, concerning which we have eleven slides, was a workman fifty-four years of age, married, of good habits and family history, so far as could be learned, with several healthy appearing children. I was called to see him by the family physician under whose care he had been for about two weeks, during which time he had been confined to the house. He gave a further history of some months of ill-health, but able to work.

Anæmic appearance was marked, but, having formerly been of rugged constitution, he was able to get out of bed and go about the house without assistance. Examination of the mouth disclosed interstitial gingivitis chiefly on lingual side of upper incisors. General condition of gums and buccal mucous membrane foul, and tongue heavily coated.

The patient was immediately removed to the hospital, and every effort made to build up the depleted system. Alcohol baths, beef peptonoids, bone-marrow, and other nourishing agents were prescribed, and these supplemented with hypodermic administration of strychnine, normal salt solution per rectum and directly into the tissues, etc.

The blood-count was as follows:

| Name. | Norma. | Conditon Present. |
|---|---|---|
| Red corpuscles | 5,000,000 | 1,704,640<br>1,500,000, August 10 |
| Hæmoglobin | 100 per cent. | 45 per cent.<br>30 per cent. |
| Corpuscle index | 1 per cent. | 1.3 per cent. |
| White cells | 7,000 | 93,100<br>105,000, August 10 |
| Polymorphonuclear neutrophiles | 70 per cent. | 8 per cent. |
| Small lymphocytes | 8 per cent. | 26 per cent. |
| Large lymphocytes | 20 per cent. | 60 per cent. |
| Remnants of leucocytes | ¼ per cent. | 6 per cent. |
| Megaloblasts | 0 | 1 to each 100 leucocytes |
| Normoblasts | 0 | 1 to each 100 leucocyte count |

Bacteriologic examination of the lesion showed staphylococcic infection.

Temperature ranged from 100° to 102°, with pulse indicative of increasing muscular weakness, both ranging higher toward the last.

Mental faculties were clear until about forty-eight hours before death, which occurred about ten days after I first saw him.

Operation was attempted for the purpose of removing the necrotic tissue that rapidly increased toward the end. An opening was made through the palate into the nares, but it was found to be dead in every direction as far as it seemed advisable to remove it. Gangrene of the soft tissues was so rapid that black masses were removed at each dressing several times daily.

The essentially interesting feature of this case to us at this time is the lesson taught by the blood-count, which indicates leukæmia, and microscopic sections of the tissues of the mouth at the seat of the oral affection, and of the important visceral organs, each of which shows practically the same areas of inflammation. By continuity of tissue or direct infection one might expect this to be the case with different portions of the intestinal tract, but the kidneys, liver, heart, and spleen are also more or less involved.

With this striking clinical picture in mind one can no longer question the bearing that conditions of the blood must have upon all of these organs in their common relation, nor can we fail to understand how functional disturbance of any one of them can produce a corresponding effect upon some one or more of the others.

Applying this then to the treatment of less grave mouth diseases, we find at once an answer to much confusing discussion with regard to pyorrhœa alveolaris, dento-alveolar abscess, tooth-pulps, etc.

And finally, to apply directly another feature of the subject, we may in this light realize more keenly the vital importance of a fuller and more far-reaching diagnosis in many apparently simple lesions of the mouth.

Having had one similar previous case in which from an inflammatory area in the vicinity of the third molar noma developed, and death resulted in about the same length of time, I was able to differentiate the condition from ulcerative stomatitis, or pyorrhœa alveolaris, each of which the oral appearance somewhat resembled.

In neither of these cases was there at any time a noticeable quantity of pus. Germicidal agents were absolutely ineffective in effort to check the tissue destructive process, and yet in both we found staphylococcus, not streptococcus as expected.

In contradistinction it is of interest to note the next feature, No. 12, as showing a vast destruction of tissue by pus from a simple dento-alveolar abscess, with profuse discharge for months, yet with record of complete recovery. No. 13 is in this class.

Group II., relating to benign and malignant growths, gives us additional food for reflection.

No. 14 is a picture of a dermoid cyst of the neck. Its history was a little confusing because of an attempted removal some years before, and what appeared to be a recurrence, but microscopic examination of the tumor after removal, and the microscopic section shown in No. 15, proved this to be a mistake, as the original operator could not have reached the growth itself at all.

No. 16 is a woman with a sarcoma. The history of the case seems to show originally a benign disease of the antrum neglected for years, but during the last few months a progressive increase of malignancy set in that was absolutely beyond surgical control. The progressive increase in malignancy may be seen in No. 18. No. 17, an older portion of the growth, reveals spindle-cell sarcoma.

Nos. 17, 18, and 19, spindle- and round-cell, an intermediate section, while the newest area of the growth gives us pigmentation of melanosarcoma, a hopelessly fatal condition owing to delay that I trust in the near future may come to be considered inexcusable.

No. 20 is a slide taken from a growth removed from one of my patients now in the hospital. She is sixty-three years of age, and has always heretofore enjoyed excellent health; family history good, and grown-up children seem to be healthy.

Chronic disease of the maxillary sinus through neglect and imperfect diagnosis, for the physician laughed at the suggestion of a serious disease, and several minor operations performed before she was referred to me, had finally resolved itself into carcinoma as the section indicates; and although extensive removal of tissue was made involving to a considerable extent both maxillæ and the vomer, with prescription for X-ray treatment, the prognosis is most unfavorable, the likelihood of recurrence being very great.

Bringing these demonstrations of ill-omen directly to bear upon practice, we conclude that no chronic inflammatory condition, however simple and harmless it may appear, should be allowed to continue without treatment; no source of long-continued irritation be left uncorrected even though of slight annoyance, and every suspicious growth or lesion concerning which the operator is in doubt should be promptly referred to some one whose constant familiarity in practice enables him to decide as to its true character.

No. 21 comes between the last preceding group and the one to follow, and in its two most important aspects refer to each. The tooth shown occupies a position in the skull in which it has been photographed, much like one found in the case of a patient of seventy-one years of age who had worn a full upper denture without knowledge of its presence, under operation for a suspicious-looking growth having its beginning in this region, which upon later examination proved to be malignant, and finally caused her death.

It is also similarly situated to one found in the jaw of a patient who was a chronic sufferer from excruciating trifacial pain for a period of seven years, and had become almost a mental as well as a physical wreck in consequence. With the picture before us we can readily understand why almost every known method of treatment which he had tried failed to give relief, but we should also keep in mind the fact that Cryer, Price, and other X-ray investigators have demonstrated that malposed, unerupted teeth are much more common than we formerly supposed; therefore these two classes of very serious affections need constant watchful care to guard against.

Group III. introduces a number of patients with characteristic nervous affections, all having a noticeable similarity of appearance, yet each one quite different, some of them diametrically opposite in character.

No. 22 represents a patient in one of the spasms of pain which he suffered at intervals of from one to two minutes for a period of five years. Correction of occlusion, the removal of a tooth-pulp, and a slight operation upon one of the branches of the submaxillary branch of the fifth nerve left him as shown in No. 23, free from pain. Observation for some two or more years after operation has confirmed the correctness of the diagnosis of these simple etiologic features.

Another patient suffered attacks of pain at intervals of a few minutes, and at times seconds, for twenty-five years.

Her family history revealed that several near relatives were affected by some neurosis, and she herself was a typical neurotic; but notwithstanding all this, resection of the inferior dental nerve and an operation for relief of a chronic mucous engorgement of the maxillary sinus gave much permanent benefit and entire relief from pain for a short period of time, with hopeful prospects of a complete cure.

Nos. 24 and 25 show a case of unilateral facial paralysis. Operation for a chronic necrotic condition of the lower jaw upon the affected side removed that diseased condition and gave a slight immediate relief to the paralysis, but his death some weeks later indicated that the true cause of his symptoms was central in character, though we have no post-mortem evidence of the nature of the brain lesion.

Surely one need have no stronger evidence than this mute testimony to bring a realization of the value of great care and discrimination in diagnosis, and of the home truth that in our special field, where the all powerful and wonderful fifth nerve presides, no etiologic factor is so small that it may with impunity be overlooked, and results are often out of all proportion to their causes when even the simplest may be vital in its importance.

Group IV.—No. 26 by illustration opens a field rapidly growing as the line of our horizon widens, with a central focal point at which the dentist, the oculist, the rhinologist, the surgeon, and the practitioners of general medicine meet in common interest.

Other slides shown were pictures of an individual at different periods of her life, the last one being at thirty years of age, after face, nose, and mouth have been corrected. These photographs have, I believe, been instrumental in accomplishing much good, having been shown and described by Dr. Nelson M. Black, of Milwaukee, with whom I co-operated in the treatment, and by myself before a number of medical and dental societies during the past few years, and bringing as they do such a striking record of almost total deafness, neurasthenia with mental derangement, a history of a sanitarium and the care of a keeper, followed by improvement of hearing, disappearance of neurotic symptoms, thirty pounds increase in flesh, with restoration to normal health, and finally marriage, which could not have been practicable under former conditions, all by widening of the dental arch and through it the maxillæ correction of occlusion, and treatment of the nose and middle ear, made possible by these simple providences.

I do not know who first tried to apply direct pressure to widen the dental arch by separation of the maxillary bones at the median suture. I have done it for more than ten years, and doubtless many others have, but I do believe that the first definite work of that character done for the specific purpose of assisting the cure of nasal, aural, and nervous affections systematically carried to a successful issue was done by Dr. Black and myself, and reported to the National Dental and other associations. I make this statement not for myself, but in justice to Dr. Black, as it is only to be expected that as the work assumes its full importance in the eyes of the profession there will be many claimants.

Doubtless your own Dr. Bogue builded better than he knew when he began his good fight for the presentation of the first molar. It lies at the very heart of this question, for arrest of development must be prenatal or postnatal. If the individual be normal, it is little less than a crime to deprive him of his birthright by giving opportunity for an artificial interference with developmental processes, and the first molar is the very key to facial and maxillary growth, erupting as it does at a stage of life so important as to be termed a period of stress, and, held accountable by Kierman for epilepsy and like affections in many cases, its importance cannot be questioned. If, on the other hand, through degenerate heredity, malnutrition, or other cause, the child be born into the world with a tendency to insufficient bone development, it is even more incumbent upon us to assist the necessarily crowded dental organs to assume a proper position through aiding the proper adjustment of those bones upon which the natural form of every facial feature depends, because in almost every such individual there is a neurotic tendency. Such a child as is here shown should be known at once as one that would require early treatment to avoid the future trouble that the history of her case describes.

All of the remaining pictures, which there is not time to describe in detail, have been treated by operation with the foregoing principles in mind, the chief effort being to restore the deformed parts to their normal relations, for, after all, cleft palate and harelip are only greater deformities due primarily to much the same causes as the lesser deformities of dental irregularity, and the same rules apply, for where forcible crushing of the bones for closure of palates in early infancy has been resorted to, this deformity in later life is often very great.

In closing I would say, let no one forget that without a full dental arch there can seldom or never be a perfect nose, nor can the associated sinuses be normal. With constriction or obstruction of the nares, there cannot be a healthful nose and throat; there must be hypertrophies and chronic thickening of the nasal mucous membrane, deviated nasal septa, spurs, atrophic, and hypertrophic rhinitis must be a natural result.

To the growing individual this means imperfect oxygenation, susceptibility to colds, throat trouble, diphtheria, lungs only partially developed, impaired resistance to pneumonia, and to that dread disease recently called the real cause of race suicide, tuberculosis, an unstable nervous system, impaired or perverted brain development.

In a final word, the sum of all this deducted from the normal record of longevity and the balance is that which it lies within our province to try to increase.

## Figures and Tables

**Figure f1:**
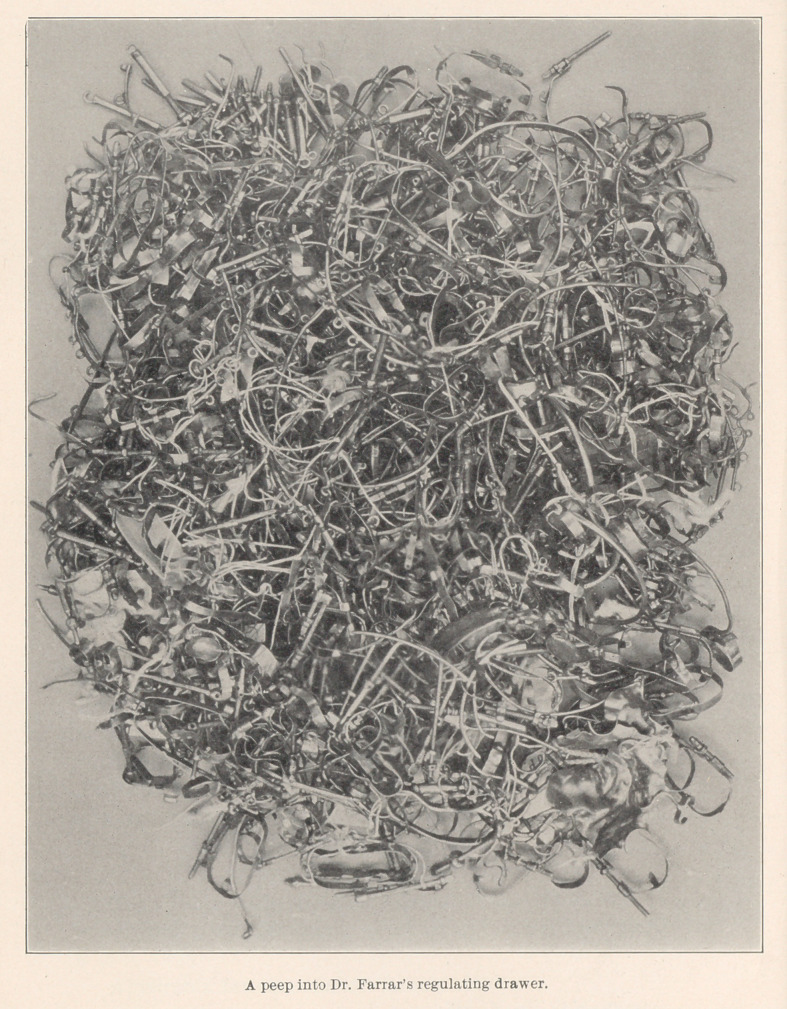


**Group I, Case 1, No. 11. f2:**
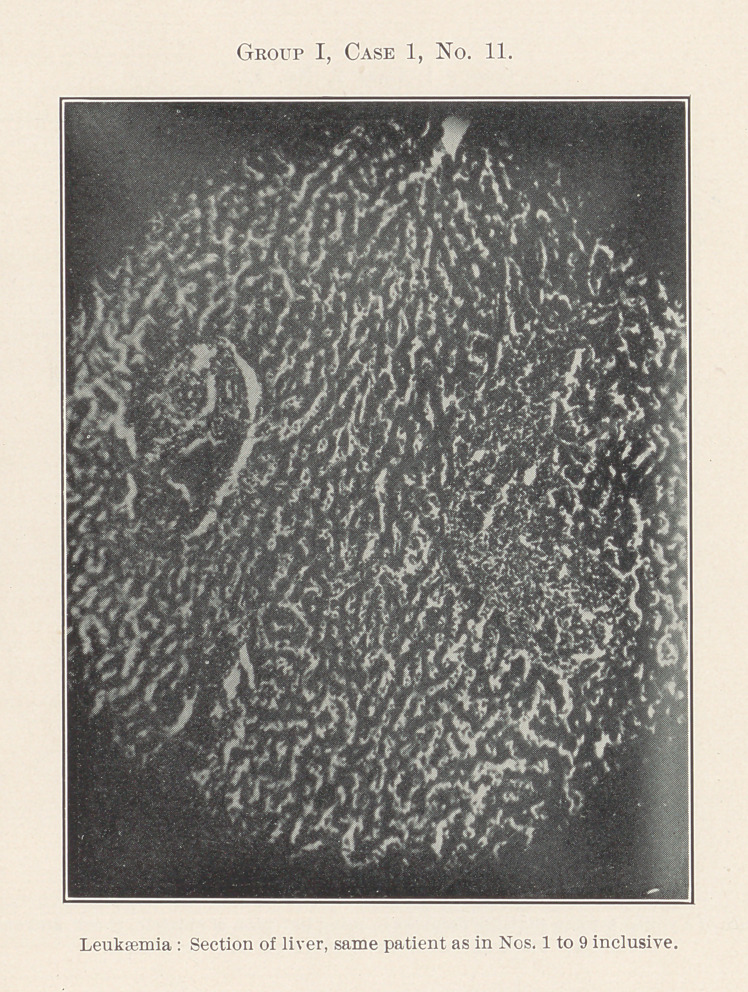


**Group I, Case 2, No. 12. f3:**
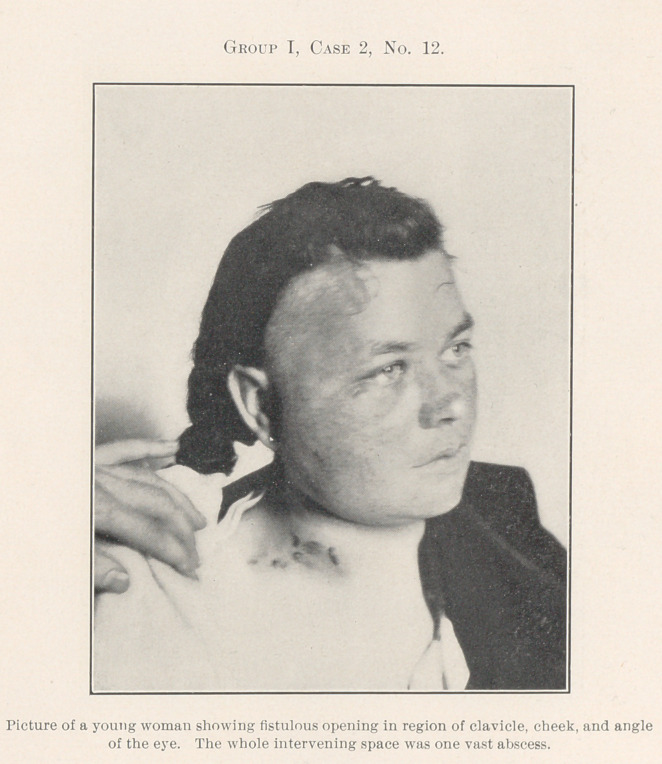


**Group I, Case 3, No. 13. f4:**
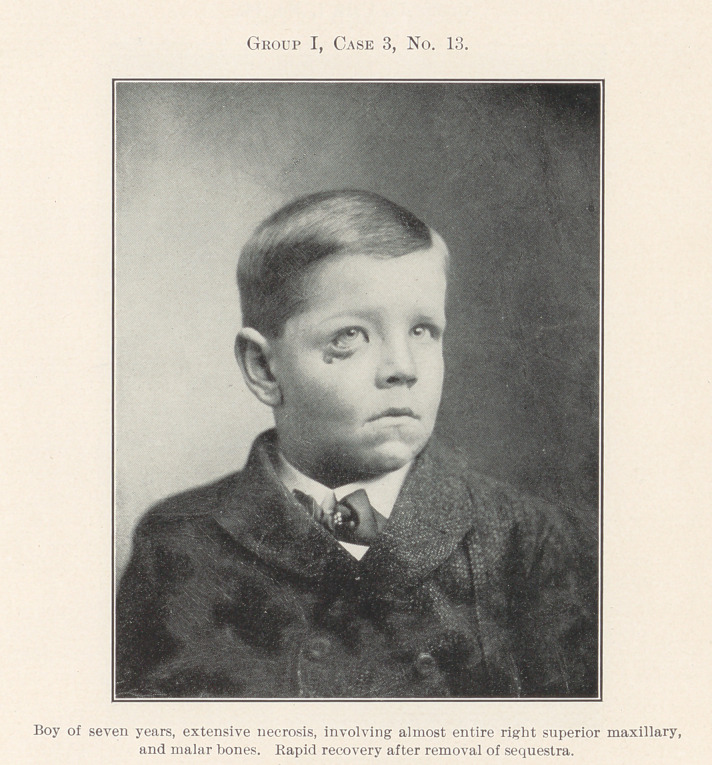


**Group II, Case 1, No. 14. f5:**
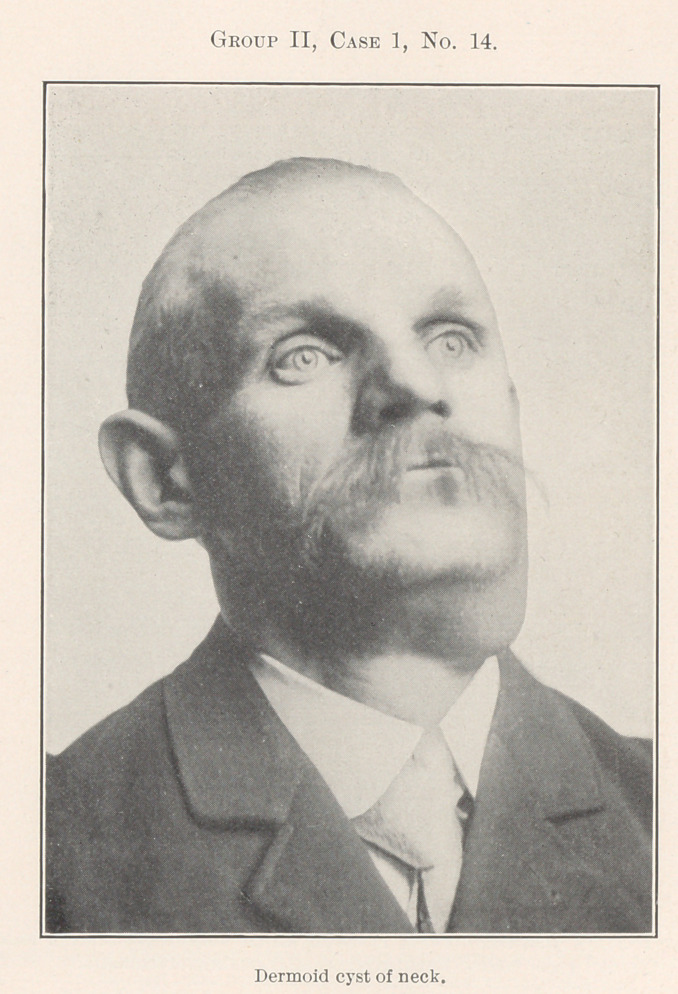


**Group II, Case 1, No. 15. f6:**
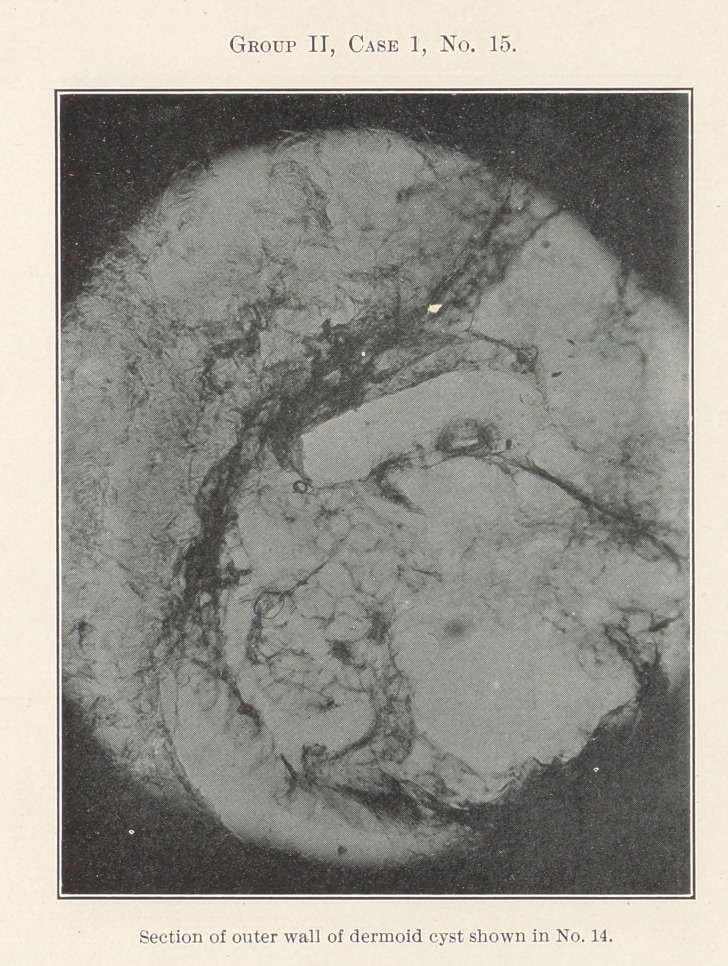


**Group II, Case 2, No. 16. f7:**
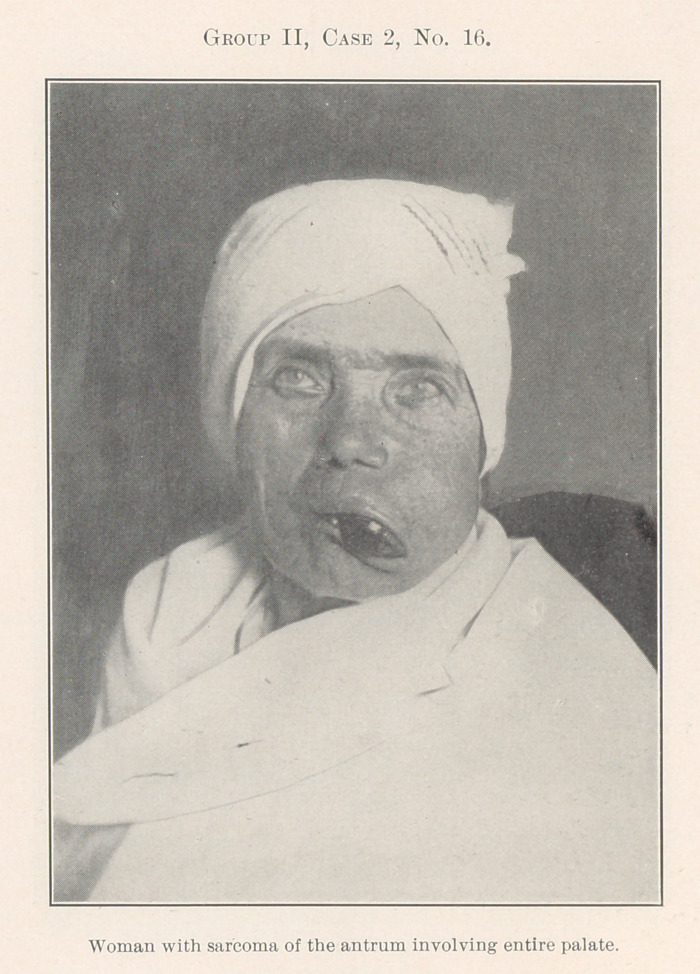


**Group II, Case 2, No. 17. f8:**
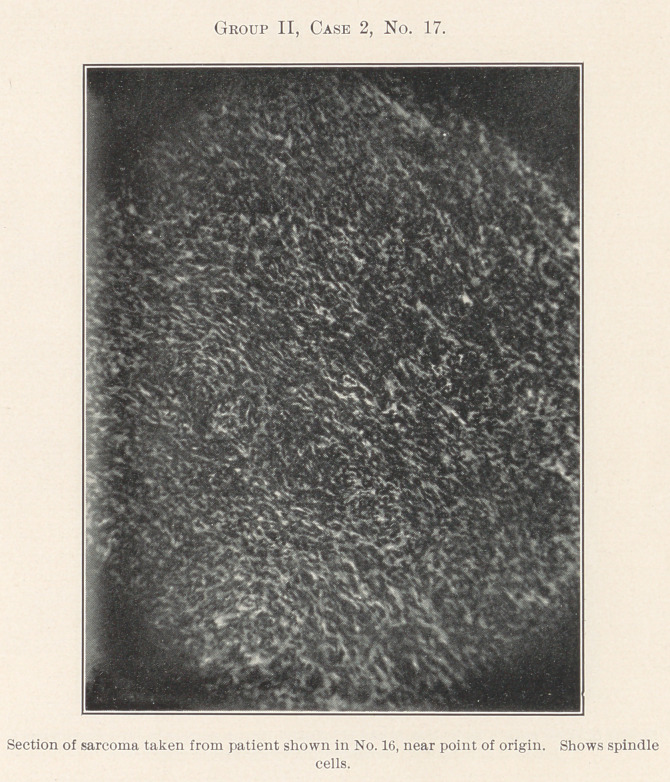


**Group II, Case 2, No. 18. f9:**
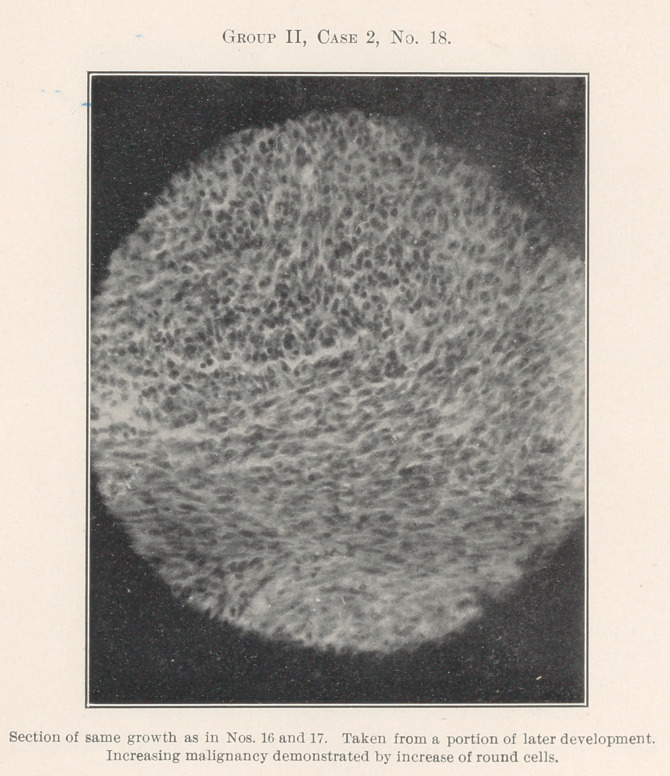


**Group II, Case 2, No. 19. f10:**
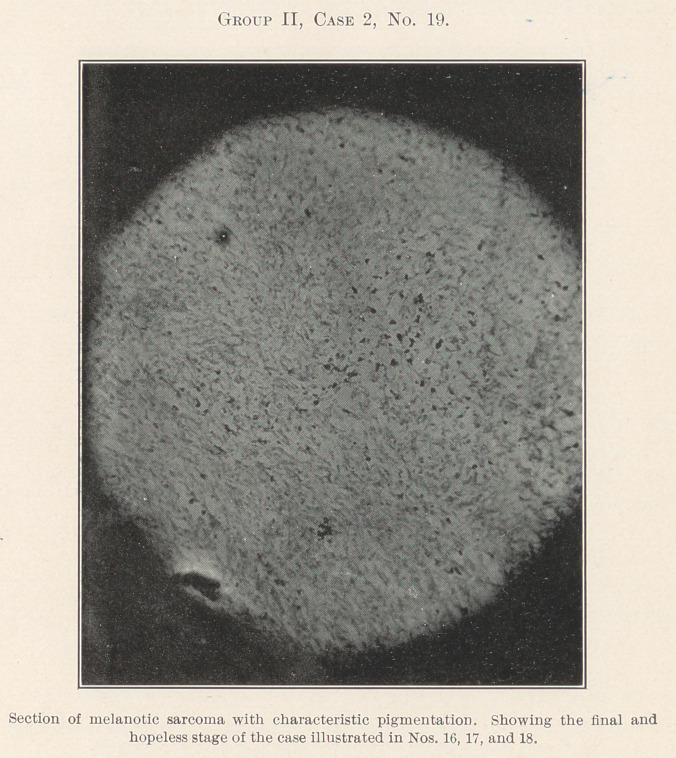


**Group II, Case 3, No. 20. f11:**
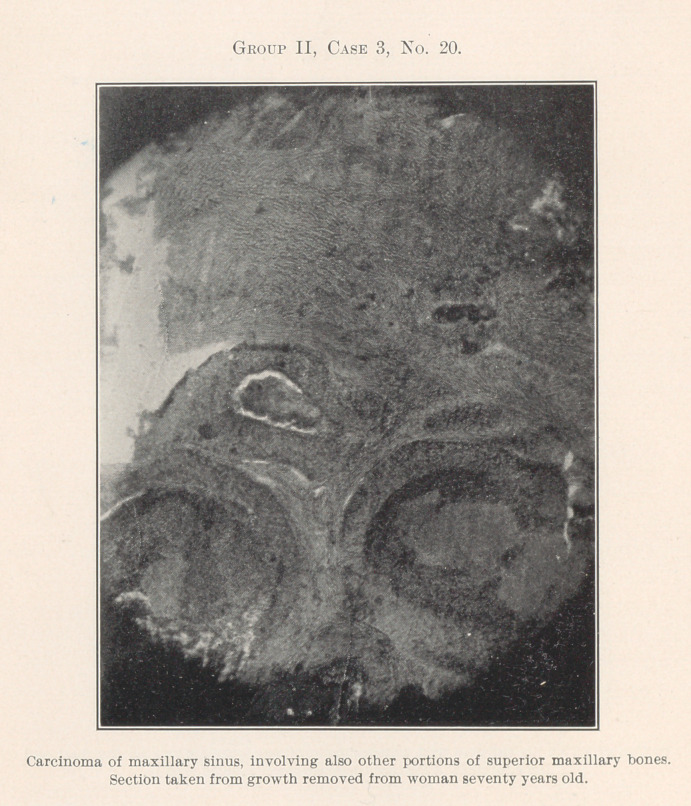


**Group I, Case 1, No. 1. f12:**
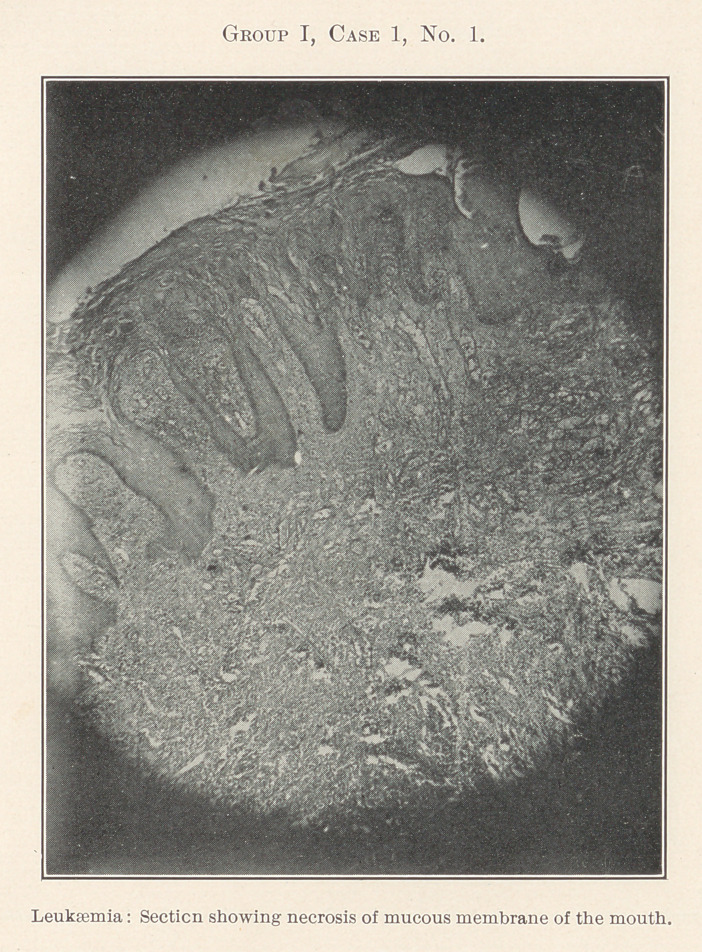


**Group I, Case 1, No. 2. f13:**
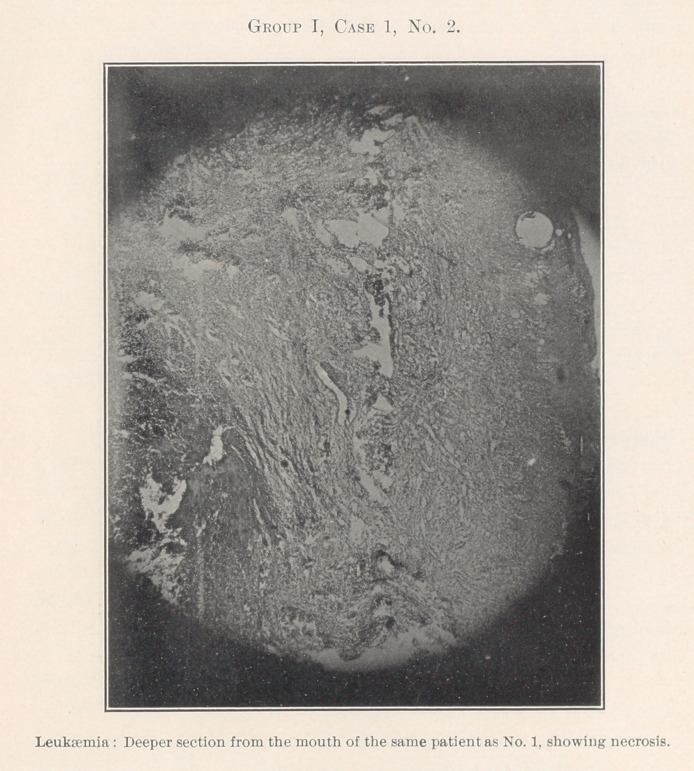


**Group I, Case 1, No. 3. f14:**
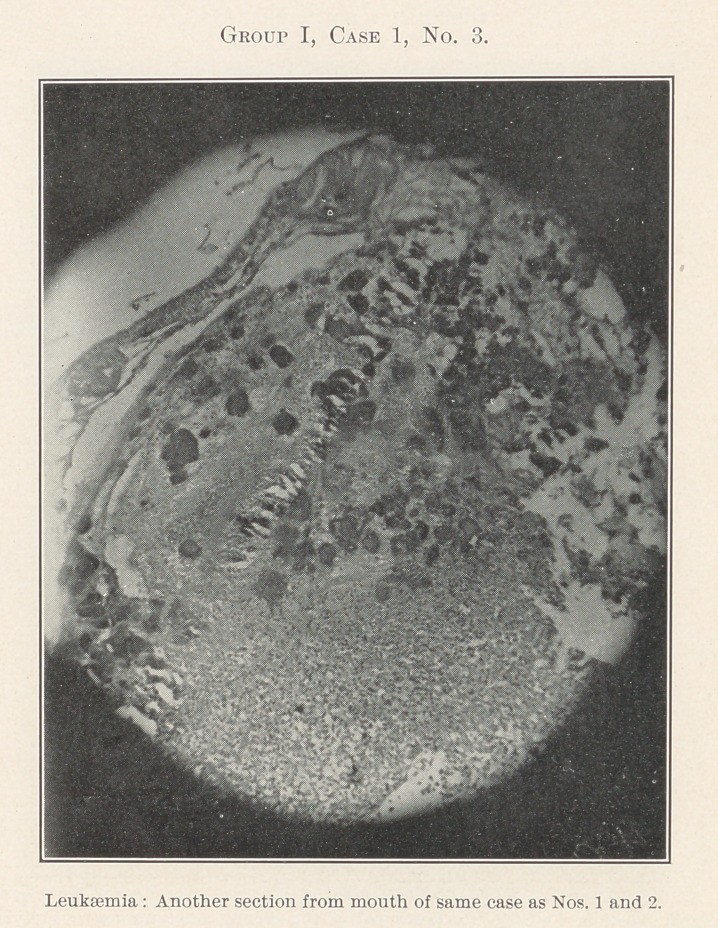


**Group I, Case 1, No. 4. f15:**
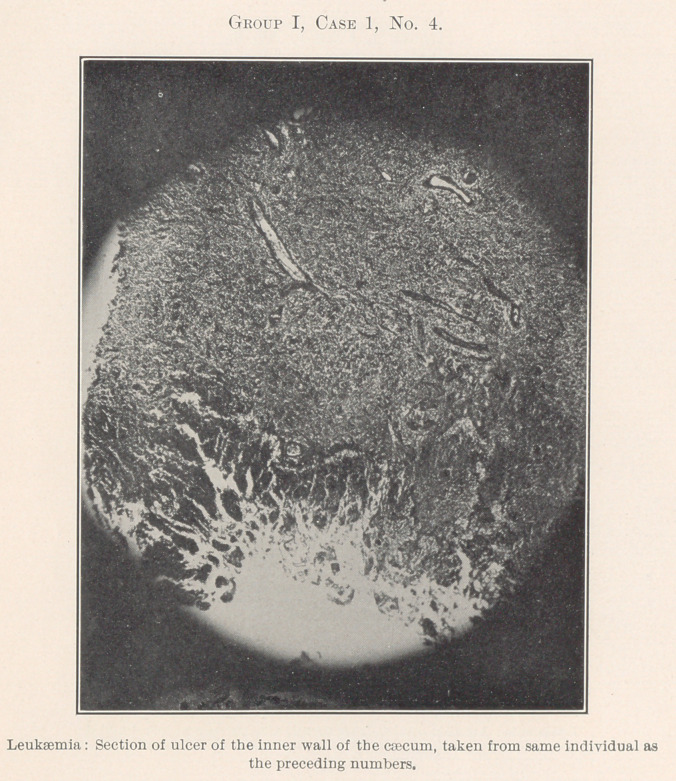


**Group I, Case 1, No. 5. f16:**
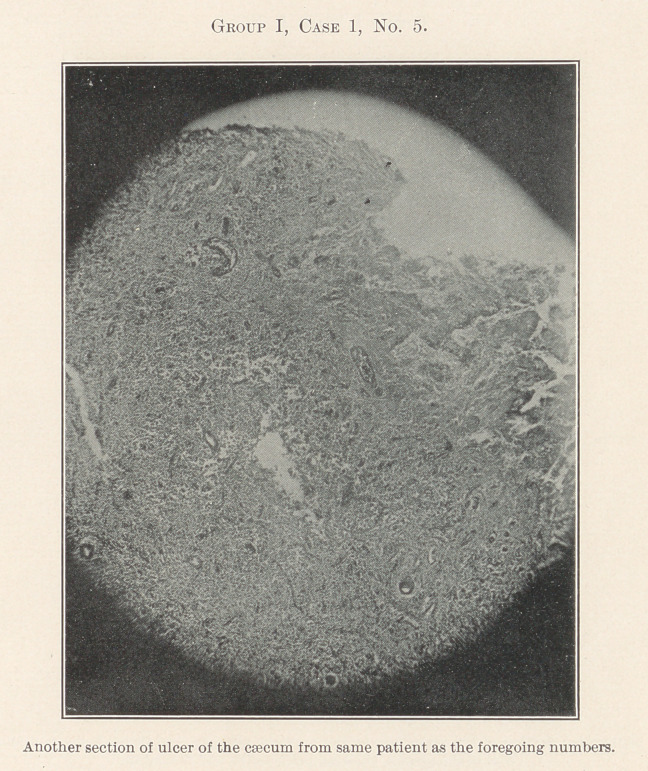


**Group I, Case 1, No. 6. f17:**
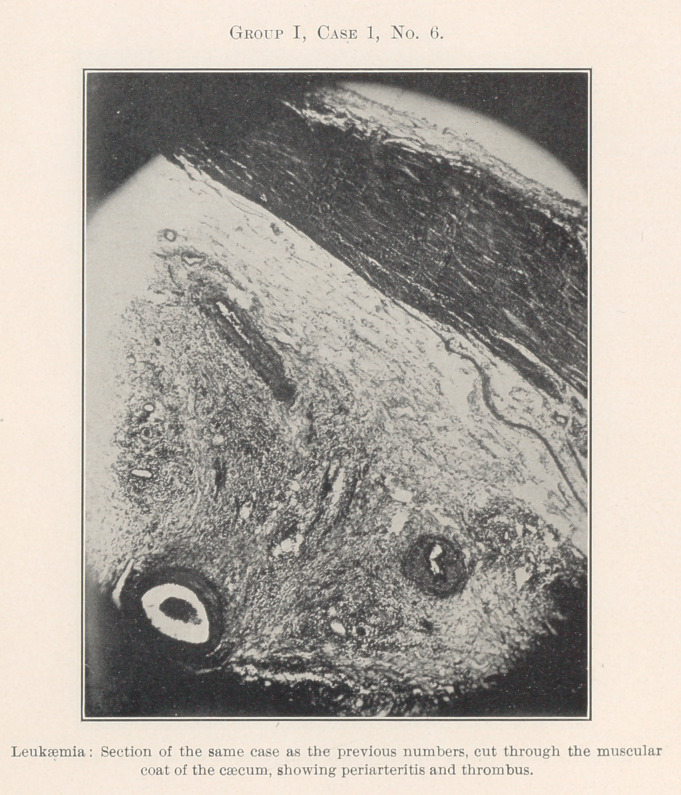


**Group I, Case 1, No. 7. f18:**
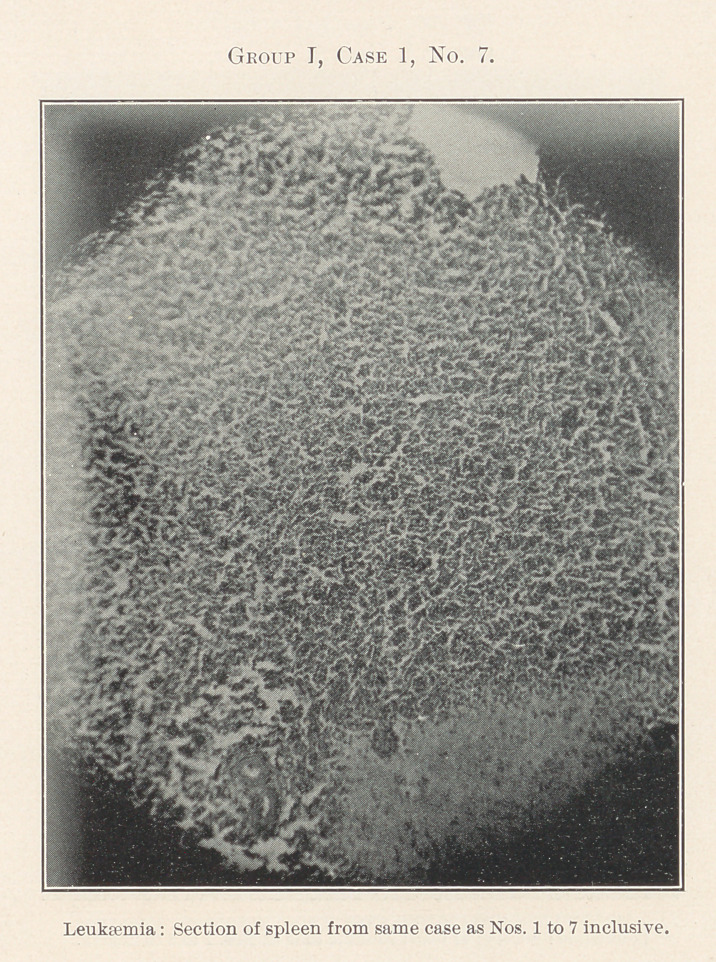


**Group I, Case 1, No. 8. f19:**
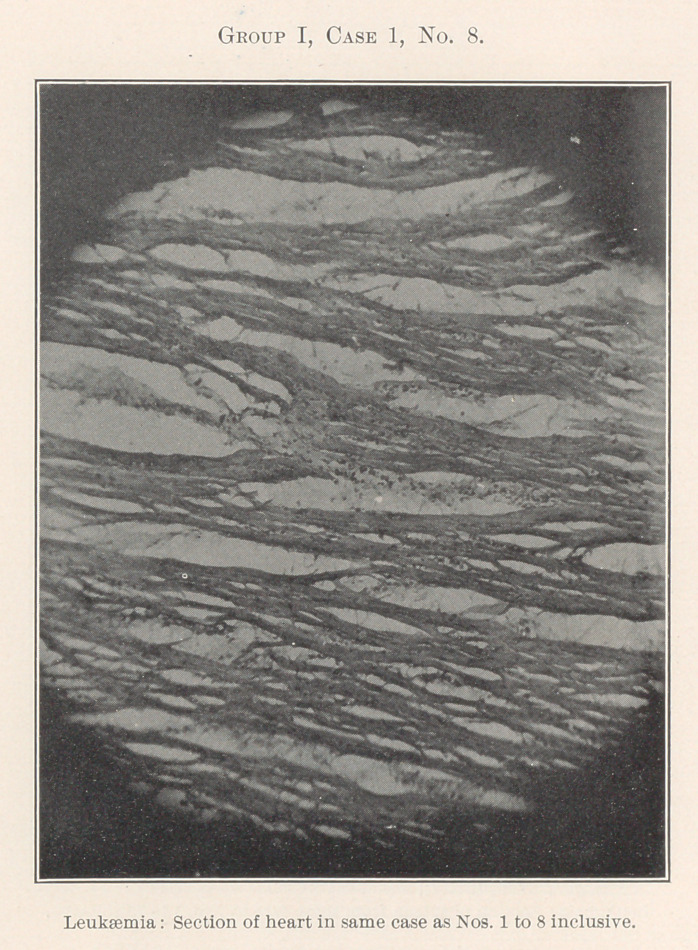


**Group I, Case 1, No. 9. f20:**
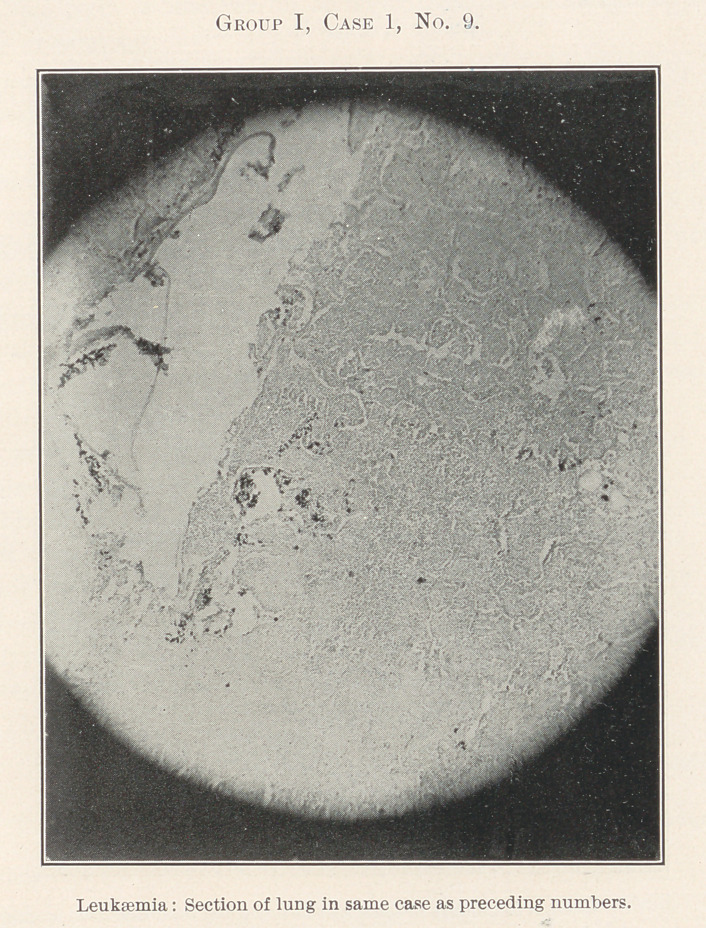


**Group I, Case 1, No. 10. f21:**
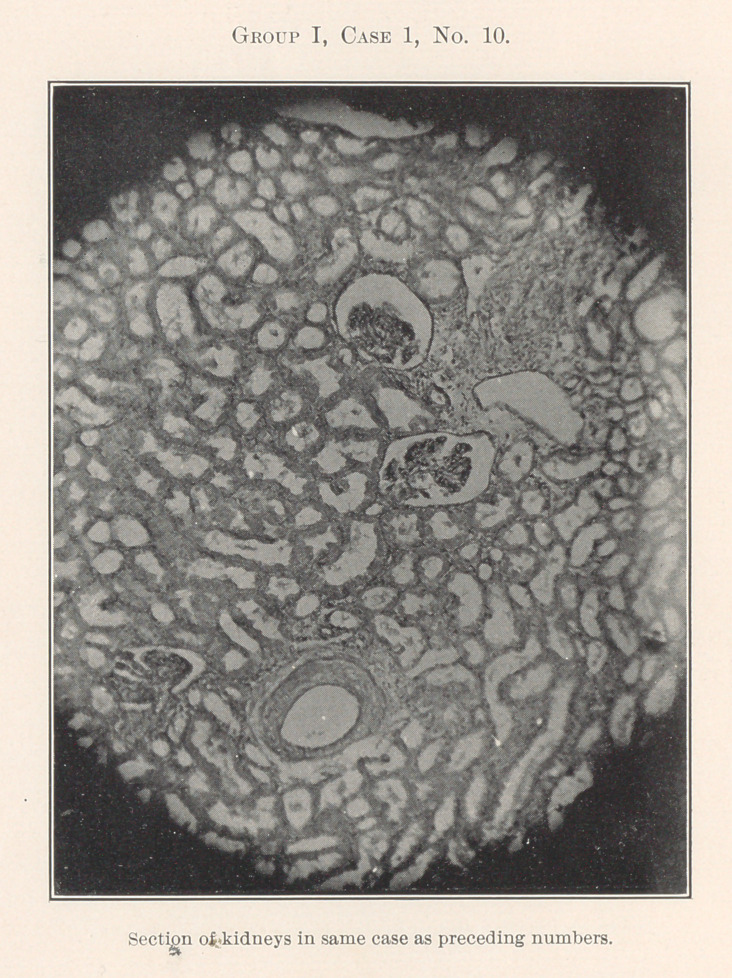


**Group II, No. 21. f22:**
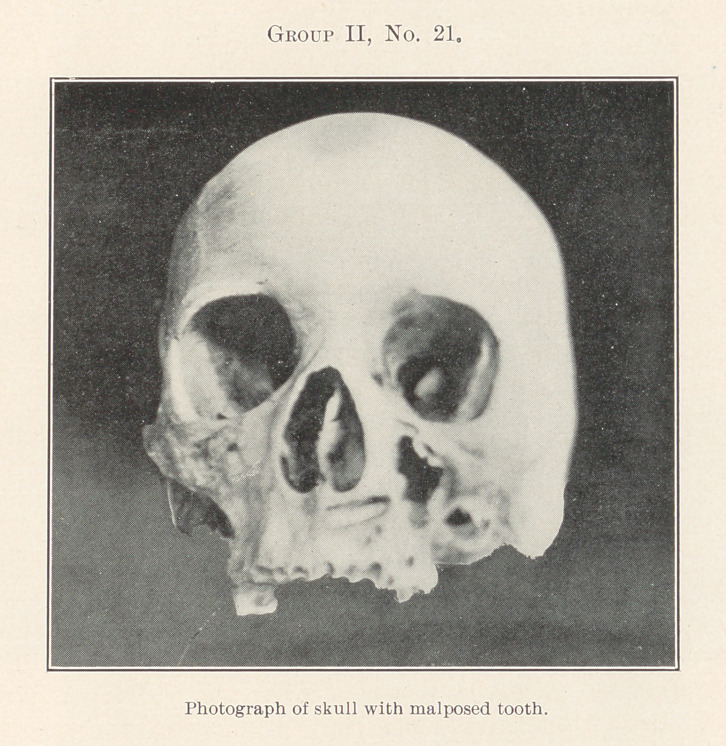


**Group III, Case 2, Nos. 22, 23. f23:**
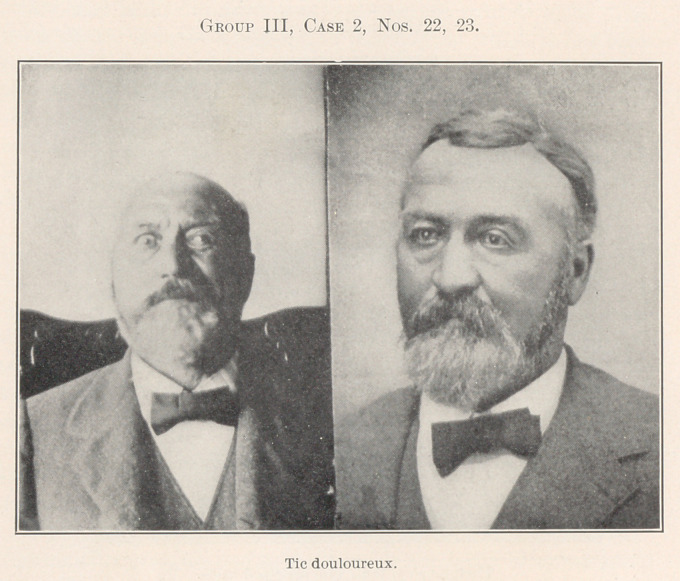


**Group III, Case 3, No. 24. f24:**
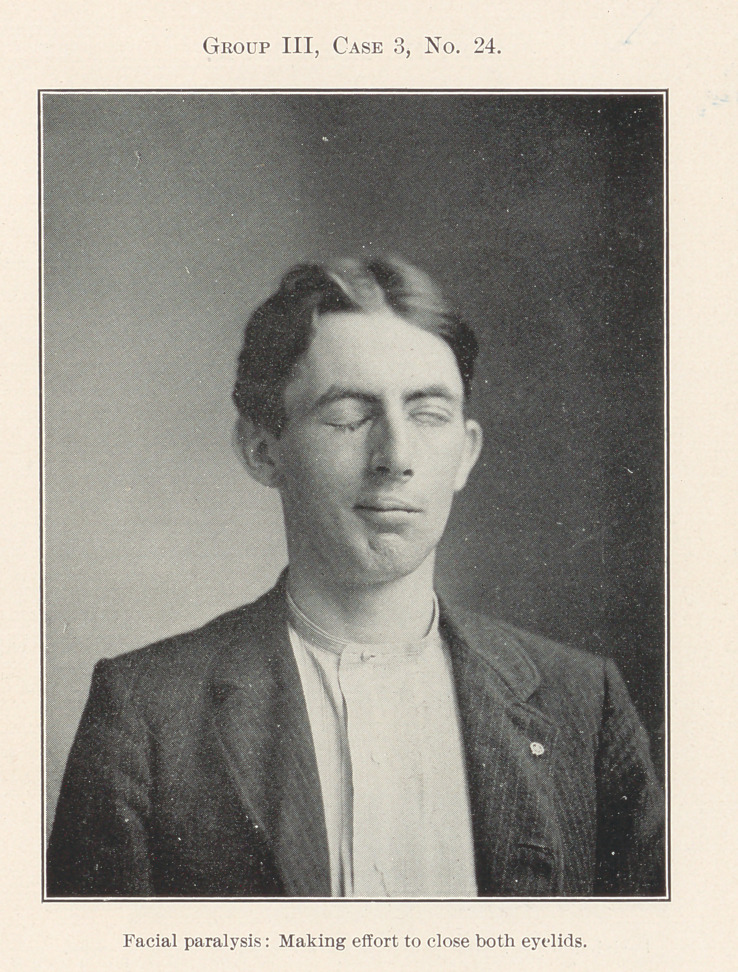


**Group III, Case 3, No. 25. f25:**
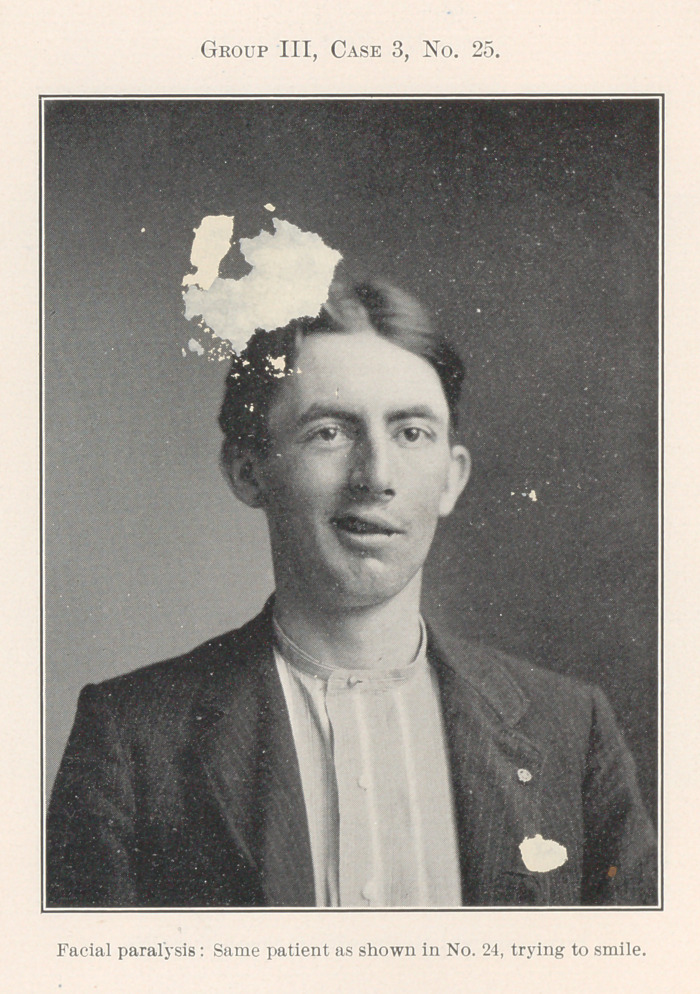


**Group IV, Case 3, No. 26. f26:**
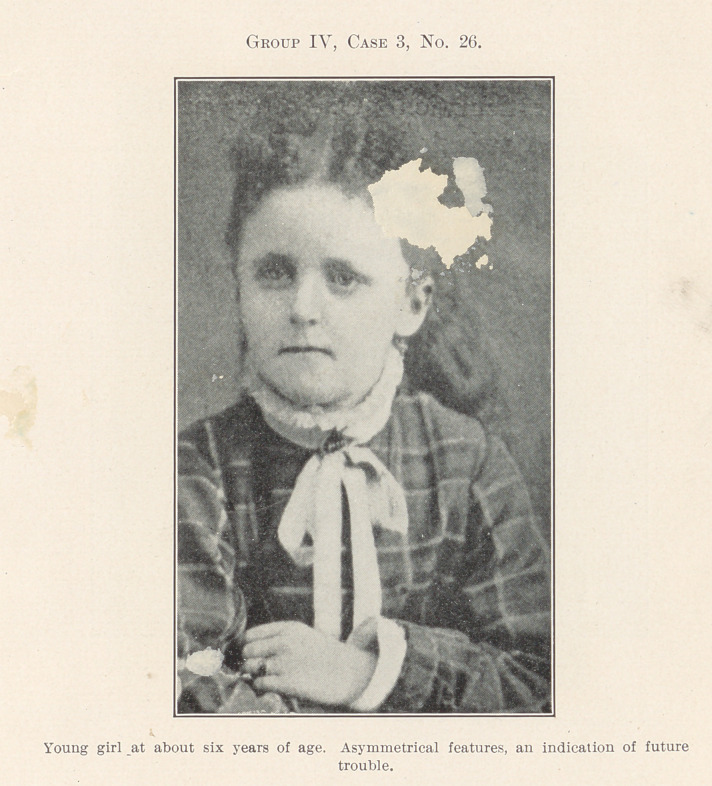


**Group V, Case 1, No. 27. f27:**
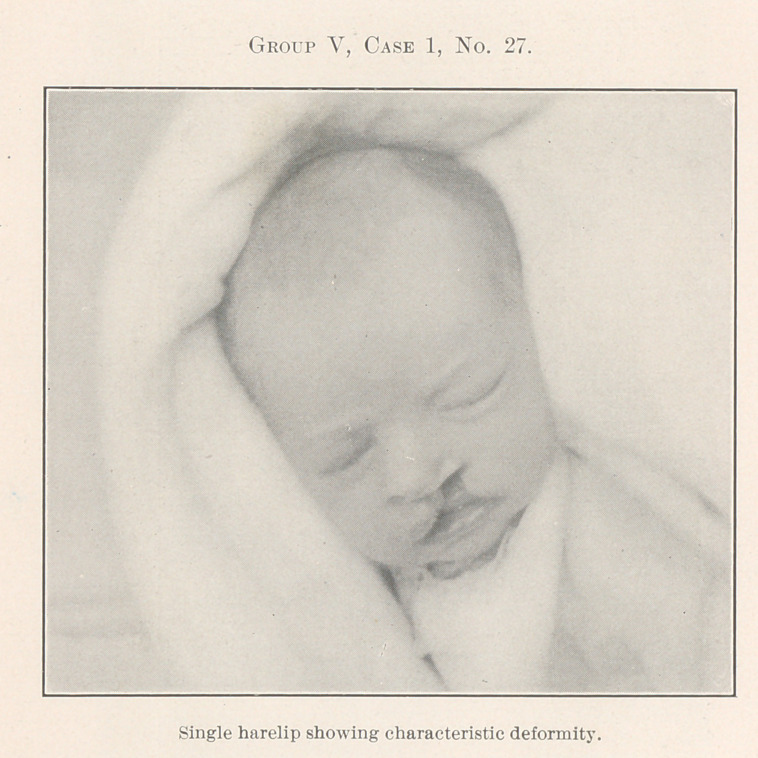


**Group V, Case 1, No. 28. f28:**
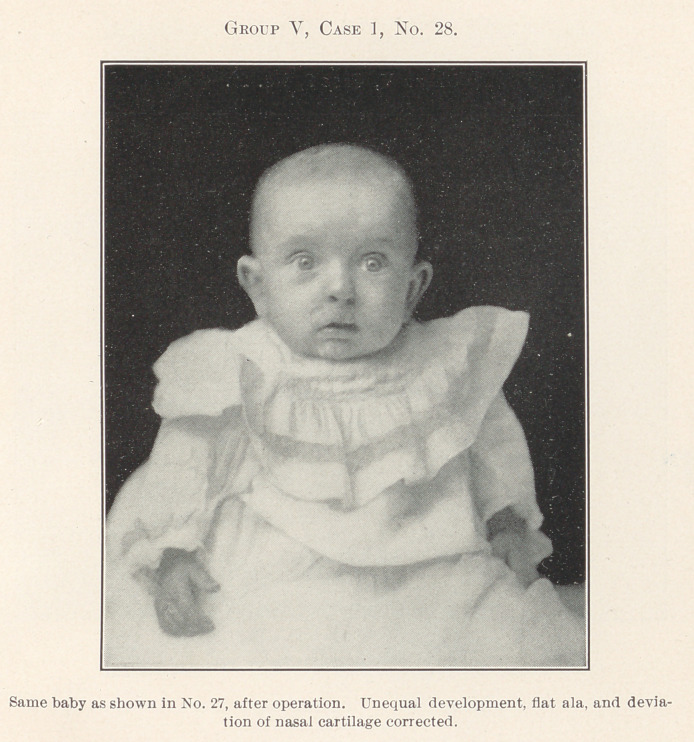


**Group V, Case 2, No. 29. f29:**
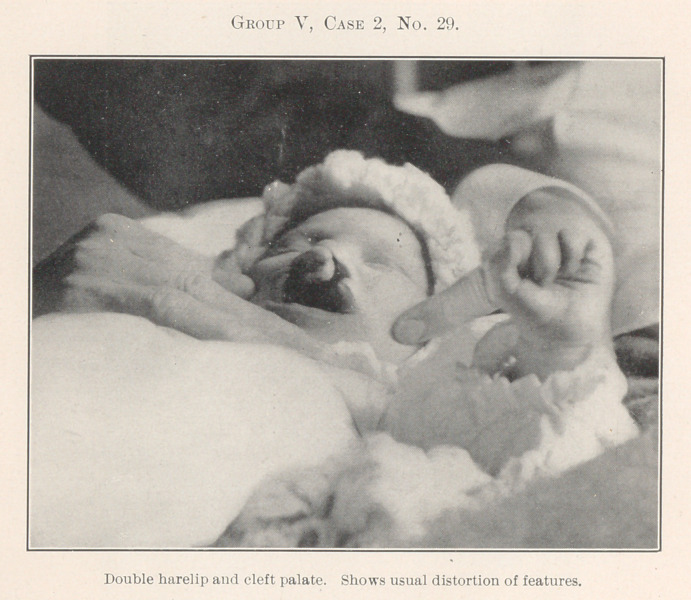


**Group V, Case 2, No. 30. f30:**